# Necrotizing Soft Tissue Infections: Intensive Care Unit (ICU) Survivor’s Long-Term Functional Outcomes and Quality of Life

**DOI:** 10.7759/cureus.77530

**Published:** 2025-01-16

**Authors:** Inês Carqueja, Carolina Tintim Lobato, António Pedro Ferreira, Ernestina Gomes

**Affiliations:** 1 Intensive Care Unit, Hospital Pedro Hispano, Matosinhos, PRT; 2 Anesthesiology Department, Hospital Pedro Hispano, Matosinhos, PRT

**Keywords:** hyperbaric oxygen therapy, long-term outcome, necrotising soft tissue infections, postoperative sequelae, quality of life

## Abstract

Introduction

Necrotizing soft tissue infections (NSTIs) are rare, rapidly progressing infections of the skin, fascia, and muscle causing necrosis, frequently requiring intensive care unit (ICU) admission. Treatment includes surgical debridement, organ support, antibiotics, and hyperbaric oxygen therapy (HBO). NSTIs require aggressive debridement, leaving survivors with wounds and functional deficits. Quality of life (QoL) is decreased in NSTI survivors, including physical and mental health. Our goal was to analyze the long-term outcomes of patients admitted to the ICU for NSTIs. We evaluated sequelae and QoL and aimed to correlate these with patient- and treatment-related factors.

Methods

All NSTI patients admitted to our ICU and treated with HBO between 2007 and 2021 were included. A review of medical records was followed by a phone interview. Demographic-, admission-, infection-, and treatment-related data were collected. Long-term outcomes analyzed included ICU/hospital mortality, one-year mortality, physical sequelae, functional capacity, and characterization of health-related QoL in 2022.

Results

Seventy-four patients were identified, 29 of which died and five were lost to follow-up. Patients were predominantly male, with a median age of 62 years. The median length of stay in ICU and hospital was 10 and 50 days, respectively. Fifty-nine (94%) patients were independent at admission (Clinical Frailty Scale (CFS) ≤4). NSTIs mainly concern the perineum or lower limbs. Most infections were polymicrobial and caused multiorgan dysfunction. The 30-day mortality was 22%, with a one-year mortality of 26%. Prevalent sequelae included hernias, scarring, and the need for intestinal ostomies. Most patients were independent on follow-up (CFS ≤ 4), denying limitations in mobility (23 patients, 61%), self-care (31 patients, 82%), or daily activities (26 patients, 68%). Chronic pain was identified in 16 patients (42%) and 13 patients (34%) reported anxiety or depression. The median value of self-perceived global health status was 72.5%. We found an association between mortality and illness severity. The small sample size regarding patient and treatment characteristics precluded further significant statistical analysis in our study.

Conclusion

NSTIs are life-threatening infections with lifelong consequences. Despite frequent physical sequelae, long-term QoL in NSTI survivors may be satisfactory, and long-term functional capacity may remain reasonable after acute disease resolution.

## Introduction

Necrotizing soft tissue infections (NSTI) are rare, rapidly progressing infections of the skin, fat, fascia, and muscle that lead to necrosis of these structures. Symptoms include disproportionate local pain, swelling, and erythema, as well as general symptoms of fever and malaise. Necrotizing infections may occur after traumatic injuries, surgical procedures, soft tissue injuries (e.g., contusions), and minor breaches of the skin or mucosa, although frequently the cause cannot be identified [1-3]. NSTIs can be divided according to the causative organisms. Type I infections are polymicrobial, including aerobic and anaerobic organisms, and are the most frequent. Type II infections are monomicrobial, commonly associated with group A Streptococcus bacteria [1,4,5].

Treatment of NSTIs includes urgent surgical debridement, organ support, and appropriate antibiotic therapy. Admission to the intensive care unit (ICU) is often required due to septic shock with multiorgan dysfunction [1,2]. Adjunctive treatment with hyperbaric oxygen therapy (HBO) may shorten the time to source control and reduce the incidence of complications [6-8]. Despite advances in critical care and surgical techniques, mortality due to these infections remains close to 20% [9].

Necrotizing infections cause considerable tissue destruction, with frequent need for aggressive surgical debridement and amputation. Survivors are often left with extensive wounds and functional deficits. Prolonged stays in the ICU are frequent and increase the risk of post-intensive care syndrome (PICS) and hospital-related complications. Quality of life (QoL) has been shown to be decreased in survivors of NSTIs, with studies demonstrating effects both on physical and mental health [10-13]. Post-discharge studies show a significant impact on perceived body image, increased insecurity, and a significant incidence of depressive and post-traumatic stress disorders [11,14].

Outcomes of NSTI survivors have mostly been assessed in small, single-center studies, with few papers focusing on long-term functional outcomes and patient-perceived QoL. The objective of this study was to analyze the long-term outcomes of patients admitted to the ICU for NSTI and treated with HBO. We aimed to correlate these outcomes with patient- and treatment-related factors.

## Materials and methods

Study design and setting

This retrospective observational study was conducted in an adult medical-surgical ICU at Hospital Pedro Hispano, Matosinhos, Portugal, and analyzed patient data over a period of 14 years (from 2007 to 2021). 

A review of medical records was followed by a phone interview with the patient or his next of kin that took place in February 2022. 

Demographic-, admission-, infection-, and treatment-related data were collected. Demographic data included age, sex, comorbidities (diabetes mellitus, obesity, arterial hypertension, heart failure, ischemic heart disease, smoking, chronic obstructive pulmonary disease, chronic renal failure, drug abuse, active neoplasia, chronic corticoid therapy, chemotherapy, and hypocoagulation), and functional capacity at ICU admission. Functional capacity was evaluated using the Clinical Frailty Scale (CFS) [15]. Admission-related data included the date of hospital/ICU admission/discharge and severity of illness scores (APACHE II and SAPS II). Infection-related data included the primary site of infection, microbiological type, microbiological isolates, and predisposing factors (penetrating or non-penetrating trauma, post-operative, breach in skin/mucosa integrity, immunosuppression, or idiopathic). Treatment-related data included the number of surgeries, number of HBO sessions, and time to source control (defined as the time between diagnosis and the last HBO session, since source control was the primary indication for HBO treatment interruption).

Long-term outcomes included ICU and hospital mortality, one-year mortality, physical sequelae, functional capacity, and QoL in 2022. QoL was characterized through the EuroQoL-5-Dimensional-3-Levels (EQ-5D-3L) questionnaire and EuroQoL-Visual Analog Scale (EQ-VAS) [16,17]. The EQ-5D-3L system includes five dimensions: mobility, self-care, usual activities, pain/discomfort, and anxiety/depression. Each dimension is classified by the patient into three levels: no problems, some problems, or extreme problems. The EQ-VAS evaluates the patient’s self-rated health on a vertical visual analog scale, where 0 is the “worst imaginable health state” and 100 is the “best imaginable health state.”

Participants

All the patients admitted to the ICU for NSTI and treated with HBO between 2007 and 2021 were included in the study. All patients contacted regarding the follow-up interview provided informed consent. There were no exclusion criteria.

Statistical analysis

A descriptive analysis of all the collected data was conducted. The categorical variables were presented as frequencies and percentages, while the continuous variables were expressed as means and standard deviations (SD) or medians and interquartile ranges (IQR) for those variables with skewed distributions. Normal distribution was checked using the Shapiro-Wilk test or skewness and kurtosis.

Comparison tests were performed to test for an association between one-year and in-hospital mortality, presence of sequelae, QoL, and the following variables: age, CFS on admission, comorbidities, time to source control, number of surgeries, ICU/hospital length of stay, and severity of illness scores (APACHE II and SAPS II). The categorical variables were compared using chi-square tests, while the continuous variables were compared using either Spearman’s correlation coefficient, Student’s t-test, or the Mann-Whitney U test, depending on the normality of the distribution.

All the reported p-values are two-tailed, with a p value <0.05 indicating statistical significance. The analyses were performed using the Statistical Package for the Social Sciences (SPSS; version 27, IBM SPSS Statistics for Windows, Armonk, NY) software.

Ethical approval

This study was approved by Hospital Pedro Hispano’s Ethics Committee prior to data collection (certificate nº133/CES/JAS, 10/2021). The patients were informed about the research, and they all signed a consent form that assured them the anonymity and confidentiality of their data would be maintained while explaining the purpose of the study and the usefulness of the predicted results.

In patients that died before follow-up occurred, dismissal of informed consent was accepted by the Ethics Committee.

## Results

**Figure 1:**
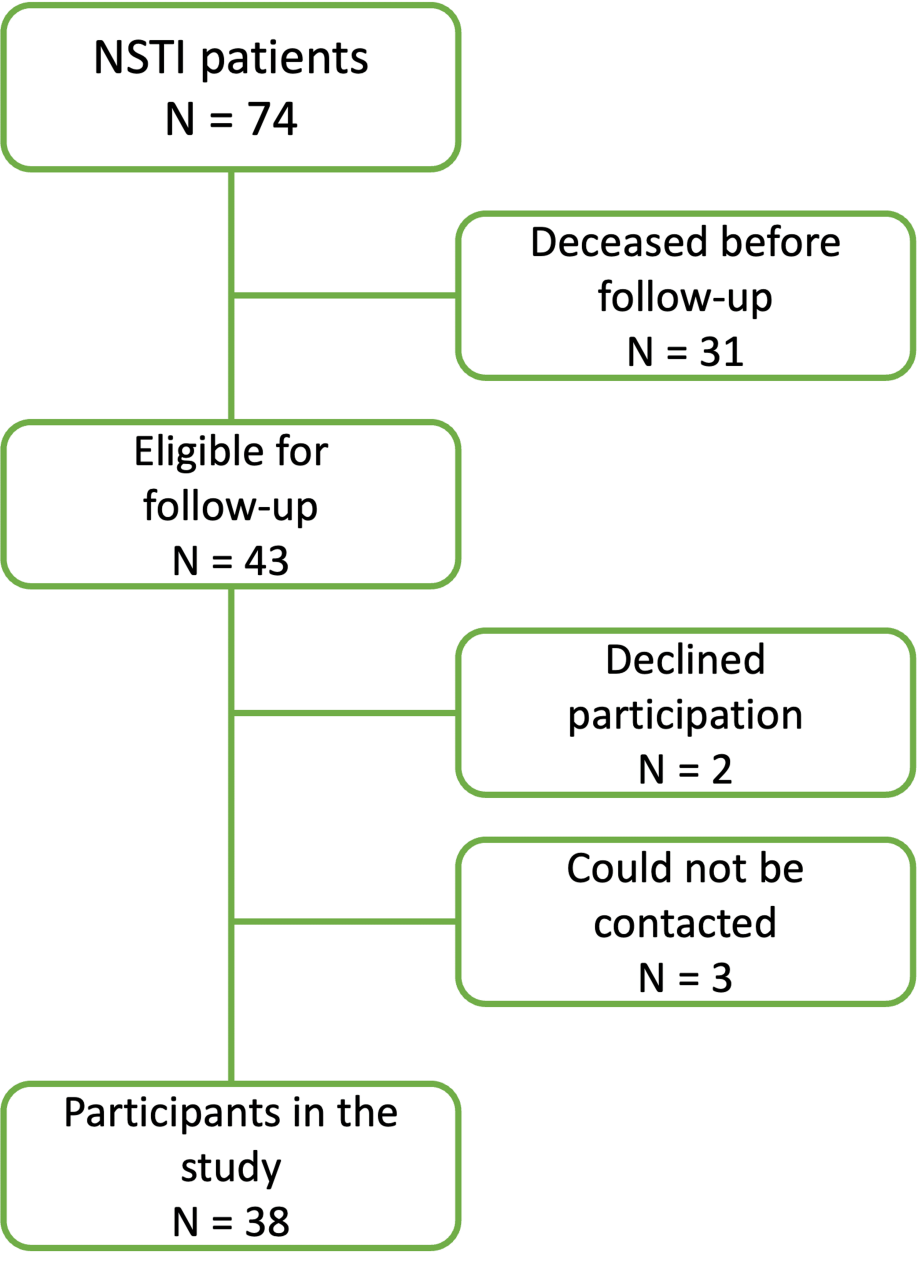
Recruitment process

A total of 74 NSTI patients were identified, of which 31 had passed away before the study was conducted. Of the 43 patients eligible for follow-up, 38 agreed to participate in the study, two declined, and three could not be contacted. A flow diagram of the study’s recruitment process is shown in Figure 1. The mean follow-up time was eight years (range: 2-15 years, SD: 3.2). We analyzed the total NSTI population and the subgroup of patients eligible for follow-up.

Participants’ characteristics

We observed predominantly male patients (67.6%), with a mean age at admission of 60 years (minimum 20 and maximum 82 years, SD: 13.7). Ninety-four percent of patients were functionally independent at admission (CFS ≤4).

A total of 38 patients were followed up. They were predominantly male (68.4%), with a mean age of 57 years. The majority were functionally independent at admission, with 97% classified as having a CFS ≤4. The most frequent comorbidities observed were arterial hypertension, obesity, and diabetes mellitus.

NSTIs mainly affect the perineum or the lower limbs, with fewer infections located in the abdomen, upper limbs, or other sites. This anatomical distribution may be influenced by the inexistence of thoracic surgery at the hospital where the study was conducted, with infections affecting the mediastinum routinely transferred to other centers. Most NSTIs originated in infections or breaches of skin/mucosa, with a smaller proportion of cases caused by post-surgical complications.

Infections were mainly polymicrobial (type I). Microbiological identification of the causal agents was possible in 90% of cases, mainly on surgical tissue samples. The majority of patients developed sepsis and multiorgan dysfunction (93.2%).

Most patients (92%) had no complications from the hyperbaric oxygen treatment. Four cases of minor and self-limited complications were observed (bronchospasm n = 2, bradycardia n = 1, psychomotor agitation n = 1). One patient suffered barotrauma with associated Eustachian tube dysfunction.

The mean time to source control was 9.2 days in the total NSTI population and 9.5 days in the follow-up group. Source control was defined as the absence of local progression of infection, determined by the attending surgical team. HBO treatments were performed daily or twice daily from admission to the moment of source control. The median number of surgeries was six in both groups, and the mean number of HBO sessions was seven in the total NSTI population and eight in the follow-up group.

The median ICU length of stay was 10 days in the total NSTI population and 12 days in the follow-up group. Median hospital length of stay was higher in the follow-up group (60 days, versus 47 days in the total population). The in-hospital mortality was 23.2%, with a 30-day mortality of 22.2% and a one-year mortality of 25.8%. Table 1 summarizes patient and treatment characteristics in both groups.

**Table 1:** Demographic-, infection-, and treatment-related data on the total NSTI and the follow-up population

Characteristics	Total NSTI population (n=74)	Follow-up population (n=38)
Age (years), mean ± SD	60 ± 13.7	57 ± 13.4
Male gender, n (%)	50 (67.6)	26 (68.4)
SAPS II score, median (p25-p75)	39 (29-50)	39 (29-50)
APACHE II score, median (p25-p75)	15.5 (10-22)	14 (9.5-20)
CFS at hospital admission, median	3	3
CFS ≤4 at hospital admission, n (%)	59 (94%)	38 (97%)
Comorbidities		
Arterial hypertension, n (%)	37 (50)	16 (42.1)
Diabetes Mellitus, n (%)	30 (40.5)	13 (34.2)
Obesity, n (%)	22 (29.7)	11 (28.9)
Smoking, n (%)	16 (21.6)	11 (28.9)
Ischemic heart disease, n (%)	7 (9.5)	3 (7.9)
Hypocoagulation, n (%)	6 (8.1)	3 (7.9)
Active cancer, n (%)	5 (6.8)	1 (2.6)
Heart failure, n (%)	4 (5.4)	1 (2.6)
Cerebrovascular disease, n (%)	4 (5.4)	1 (2.6)
Chronic obstructive pulmonary disease, n (%)	3 (4.1)	1 (2.6)
Corticotherapy, n (%)	3 (4.1)	1 (2.6)
Drug abuse, n (%)	2 (2.7)	1 (2.6)
Chronic renal failure, n (%)	2 (2.7)	0 (0)
Chemotherapy, n (%)	2 (2.7)	0 (0)
Location of NSTI		
Perineum, n (%)	28 (37.8)	15 (39.5)
Lower limb, n (%)	21 (28.4)	10 (26.3)
Abdomen, n (%)	10 (13.5)	5 (13.2)
Upper limb, n (%)	5 (6.8)	2 (5.3)
Abdomen and perineum, n (%)	3 (4.1)	1 (2.6)
Retroperitoneum, n (%)	2 (2.7)	2 (5.3)
Perineum and lower limb, n (%)	2 (2.7)	1 (2.6)
Multiple foci, n (%)	2 (2.7)	2 (5.3)
Neck, n (%)	1 (1.4)	0 (0)
Predisposing factor for NSTI		
Local infection/breach of mucosa, n (%)	39 (52.7)	18 (47.4)
Post-surgical, n (%)	13 (17.6)	7 (18.4)
Post-traumatic, n (%)	5 (6.8)	3 (7.9)
Intravenous drug use, n (%)	2 (2.7)	1 (2.6)
Unknown, n (%)	13 (17.6)	9 (23.7)
Treatment characteristics		
Polimicrobial infection, n (%)	44 (62.9)	23 (60.5)
Multiorgan dysfunction, n (%)	69 (93.2)	34 (89.5)
ICU length of stay (days), median (p25-p75)	10 (6-19)	12 (8-19)
Hospital length of stay (days), median (p25-p75)	47 (20-73)	60 (29-83)
Time to source control (days), mean ± SD	9.2 ± 5.4	9.5 ± 5.4
Number of surgeries, median (p25-p75)	6 (1-11)	6 (2-10)
Number of HBO sessions, mean ± SD	7.2 ± 4	8 ± 4

Descriptive data concerning the outcomes of interest

On follow-up, the majority of patients (84%) reported being currently independent (CFS ≤4). Twenty-one percent of all patients denied any active disease symptoms (CFS ≤2). The median CFS score on follow-up was 3.

Most patients (95%) reported some sort of physical sequelae related to the NSTI. The most common sequelae were abdominal hernias, significant scarring, and intestinal ostomies. Table 2 summarizes the data concerning NSTI sequelae.

**Table 2:** Long-term outcomes observed in the follow-up population

Characteristics	Follow-up population (n=38)
CFS on follow-up, median (p25-p75)	3 (3-4)
Sequelae	
Abdominal hernia, n (%)	15 (39.5)
Intestinal ostomy, n (%)	14 (36.8)
Significant scarring, n (%)	13 (34.2)
Impaired limb mobility, n (%)	11 (28.9)
Chronic pain, n (%)	8 (21.1)
Sexual dysfunction, n (%)	4 (10.5)
Reconstructed digestive tract, n (%)	4 (10.5)
Constipation, n (%)	4 (10.5)
Fecal incontinence, n (%)	3 (7.9)
Urinary incontinence, n (%)	3 (7.9)
Nervous injury, n (%)	3 (7.9)
Limb amputation, n (%)	2 (5.3)
Cystostomy, n (%)	1 (2.6)
Chronic fatigue, n (%)	1 (2.6)
No sequelae, n (%)	2 (5.3)

The results of the EQ-5D-3L questionnaire are summarized in Table 3. The majority of patients reported no limitation in mobility, self-care, or daily activities. Most patients reported no complaints of pain/discomfort and denied feelings of anxiety or depression. At follow-up, most NSTI survivors considered their health status to be worse or similar to that previous to the hospitalization. The median score for self-perceived global health status in the EQ-VAS was 72.5.

**Table 3:** Results of the EQ-5D-3L questionnaire in the follow-up population

EQ-5D-3L questionnaire results		
Mobility, n (%)	No limitation	23 (60.5)
	Moderate limitation	14 (36.8)
	Bedridden	1 (2.6)
Self-care, n (%)	No limitation	31 (81.6)
	Moderate limitation	4 (10.5)
	Inability to perform	3 (7.9)
Daily activities, n (%)	No limitation	26 (68.4)
	Moderate limitation	8 (21.1)
	Inability to perform	4 (10.5)
Pain/discomfort, n (%)	No pain	22 (57.9)
	Moderate pain	12 (31.6)
	Extreme pain	4 (10.5)
Anxiety/depression, n (%)	No symptoms	25 (65.8)
	Some symptoms	11 (28.9)
	Extreme anxiety/depression	2 (5.3)
Global health status, n (%)	Better	7 (18.4)
	Equivalent	13 (34.2)
	Worse	18 (47.4)
EQ-5D-3L VAS, median (p25-p75)	72.5 (55-86.3)	

Univariate analysis

There was a statistically significant association between in-hospital mortality and the SAPS II score on admission, with a mean score of 40.3 in survivors and 52.5 in the patients who died during hospitalization (p=0.042). An association was also observed between in-hospital mortality and the APACHE II score, with a mean score of 15 in the surviving patients and 22.9 in the patients who died during hospitalization (p<0.001). ICU and hospital length of stay had a statistically significant association with mortality. The median ICU length of stay was 11 days in the surviving patients and eight days in the deceased patients (p=0.003). The median hospital length-of-stay of 67 days in the surviving patients and 11 days in the deceased patients (p<0.001). The small sample size for the different patient and disease related factors precluded significant statistical analysis.

## Discussion

Our primary finding was that, contrary to the results of previous studies [10,11], health-related QoL was not significantly diminished among survivors of NSTI. Interestingly, the EQ-VAS scores in our study were comparable to those reported for general, non-ICU populations within the same age group [18].

Despite the significant number of patients with sequelae from the NSTI, we found that these do not seem to be determinants for a decreased long-term QoL. Even though the overall health status was reported to be worse compared to the pre-NSTI status in a significant proportion of patients, most did not report problems in the EQ-5D-3L domains. We believe that a standardized, validated analysis of QoL using tools such as EQ-5D-3L is very useful to ascertain the true impact of severe, mutilating diseases such as NSTIs in the long-term outcomes of ICU survivors.

Only a small number of studies evaluating QoL in NSTI patients report the long-term physical sequelae of these infections [13,19]. A long-term qualitative study by Suijker et al. reported scars and functional impairment in all patients interviewed, as well as high rates of fatigue and sleep problems [19]. A follow-up study of Fournier’s gangrene patients reported long-term complaints of high rates of sexual dysfunction, negative impact of scarring on body image, perineal discomfort, need for intestinal ostomy, and urinary symptoms [13]. Despite the large sample of patients with perineal NSTIs in our study, most reported sequelae were related to abdominal hernias and ostomies. This might be related to extensive debridement surgeries as part of treatment, including the frequent need for derivative intestinal ostomies for perineal protection. The data collection process (phone interview), allied with the cultural characteristics of the Portuguese population, may contribute to a low rate of disclosure of sexual dysfunction/complaints in our population. We propose that the use of an anonymous data collection method might have resulted in a higher incidence of reported sequelae in this area.

In our study, the low number of patients with no sequelae precluded relevant statistical analysis of the relation between patient characteristics and the occurrence of sequelae. This is a limitation of our study.

Evaluation of QoL in NSTI patients may be performed in multiple different ways. Some studies report qualitative data [19,20], while others use tools such as Medical Outcomes Short Form 36 (SF-36), Derriford Appearance Scale (DAS), Hospital Anxiety and Depression, Impact of Event Scale-Revised, and Activities of Daily Living scores [11,13,21,22]. A study on NSTIs of the upper limb by Nawijn et al. reported a median EQ-VAS score of 77, a result comparable to our findings (median 72.5), even though they included non-ICU patients. In these authors’ study, intravenous drug abuse, opioid abuse, and longer hospital length of stay were associated with a lower EQ-VAS score [22]. A study by Urbina et al. reported a self-assessed QoL median of 65 on a scale from 1 to 100. In their population, only previous cardiac disease was associated with a lower QoL on follow-up, and ICU admission was associated with a worse mental QoL [11].

Our study results could be enriched through the use of additional tools for the evaluation of QoL. Even though the EQ-5D-3L is a recognized and validated tool, the assessment of a greater number of domains and determinants of QoL could allow for the detection of the impact in specific areas of QoL that do not translate into impairment in mobility, personal care, daily activities, pain, or depression. We believe that further research could benefit from including EQ-5D-3L along with more extensive tools, such as SF-36 or DAS, for a more thorough characterization of long-term impact in QoL. The sample size and heterogeneity regarding the different variables (with small numbers for some of them) lead to difficulty in obtaining statistically significant results.

Our population of NSTI patients was similar to other series described in the literature regarding age and gender distribution [5,11,13,23,24]. The most frequent comorbidities in our analysis were arterial hypertension, diabetes mellitus, and obesity, in accordance with the findings of other authors [11,13,21,23,25]. The association between comorbidities and mortality is inconsistent in the literature. While some studies report correlations between risk factors such as cardiovascular disease and mortality [13,26,27], others found no significant correlation between comorbidities and mortality [5,10,11]. In our analysis, the reduced sample size precluded significant statistical analysis regarding the association between comorbidities, risk factors, and the outcomes of interest.

In-hospital mortality in our population was 23,2%, in accordance with other series of NSTI patients [3,10,23,24,27]. Our study included only patients admitted to the ICU, leading to a selection bias towards the most severe cases of NSTIs. Higher severity on admission (assessed by APACHE II and SAPS II scores) was associated with an increased mortality rate, consistent with findings of other studies [28,29].

Our NSTI population had a prolonged hospital length of stay (median of 47 days), reflecting the extensive period required for the treatment of these infections, frequently involving multiple surgical interventions. Literature is heterogeneous concerning hospital and ICU length of stay, with some papers comparable to our findings [5,10,13,30] and others reporting lower admission duration [22,23,27]. Shorter hospital length of stay can be explained by the inclusion of non-critically ill patients in several studies. We believe that the variability in ICU length of stay can be partially attributed to differences in local organizations regarding the level of care.

Our study has several limitations. The retrospective and monocentric character of the analysis should be taken into consideration. The very low number of patients with no reported sequelae limits the analysis and generalizability of data, due to the lack of statistical power. Despite the high proportion of responders among survivors, some results may be influenced by the relatively small sample size. The bias caused by losses of follow-up should also be taken into consideration. QoL has many determinants, and it is challenging to ascertain the true impact of the NSTI in long-term overall QoL in patients with other comorbidities.

## Conclusions

In conclusion, our study shows that NSTIs have a long-term and lifelong impact on ICU survivors, with a high prevalence of physical sequelae. We have shown that, despite the impact of several determinants of QoL, a high percentage of patients have no limitations in the EQ-5D-3L domains, and the self-perceived overall QoL is good. We believe that this emphasizes the importance of high-quality critical care and the high potential for recovery with good QoL in this subgroup of patients.

Further studies are necessary to validate these results and find potential determinants of long-term QoL that can be modifiable during the acute disease or follow-up of this population.
